# Supplementary material for: AISMPred: A Machine Learning Approach for Predicting Anti-Inflammatory Small Molecules
Source: Pharmaceuticals (Basel). 2024 Dec 15;17(12):1693. doi: 10.3390/ph17121693 (PMC11676721; doi:10.3390/ph17121693)
Supplement: Supplementary file 1 [file pharmaceuticals-17-01693-s001.zip › pharmaceuticals-3299674-supplementary.pdf]

Supplementary Materials:

Table S1: Hyperparameters of ML classifiers using training dataset.

Classifiers	Parameters	Optimal Value
**RF**	n_estimators	200
	max_depth	15
	min_samples_split	10
**ET**	n_estimators	400
	max_depth	20
	min_samples_split	5
	random_state	101
**KNN**	n_neighbors=5	5
	weights	distance
	algorithm	auto
	leaf_size	30
	p	1
**LR**	penalty	L2
	C	0.1
	dual	False
	tol	0.0001
	fit_intercept	True
	intercept_scaling	1
	class_weight	None
	random_state	None
	solver	lbfgs
	max_iter	1500
	multi_class	auto
	verbose	0
	warm_start	False
	n_jobs	None
	l1_ratio	None
**Ensemble (RF+ET)**	n_estimators	200
	max_depth	15
	min_samples_split	10
	n_estimators	400
	max_depth	20
	min_samples_split	5
	random_state	101
